# Validation and Reliability of the Alzheimer’s Disease-Commonwealth Scientific and Industrial Research Organisation Food Frequency Questionnaire

**DOI:** 10.3390/nu12123605

**Published:** 2020-11-24

**Authors:** Samantha L. Gardener, Philippa Lyons-Wall, Ralph N. Martins, Stephanie R. Rainey-Smith

**Affiliations:** 1 School of Medical and Health Sciences, Edith Cowan University, Joondalup, WA 6027, Australia; s.gardener@ecu.edu.au (S.L.G.); p.lyons-wall@ecu.edu.au (P.L.-W.); ralph.n.martins@gmail.com (R.N.M.); 2 Australian Alzheimer’s Research Foundation, Sarich Neuroscience Research Institute, Nedlands, WA 6009, Australia; 3 School of Biomedical Sciences, Faculty of Medicine and Health Sciences, Macquarie University, Macquarie Park, NSW 2109, Australia; 4 School of Psychological Science, University of Western Australia, Crawley, WA 6009, Australia

**Keywords:** validation, food frequency questionnaire, diet, Alzheimer’s disease, Australia, FFQ, weighed food record, reliability

## Abstract

Accuracy in measuring intake of dietary constituents is an important issue in studies reporting the associations between diet and chronic diseases. We modified a Commonwealth Scientific and Industrial Research Organisation (CSIRO) food frequency questionnaire (FFQ) to include foods of interest in the field of Alzheimer’s disease (AD) research. The aim of the current study was to determine the reliability and validity of the AD-CSIROFFQ in 148 cognitively normal older adults. The AD-CSIROFFQ was completed before and after completion of a four-day weighed food record. Of the 508 food and beverage items reported, 309 had sufficient consumption levels for analysis of reliability. Of the 309 items, over 78% were significantly correlated between the two questionnaire administrations (Spearman’s rank correlations). We used two additional methods to assess absolute nutrient intake agreement between the AD-CSIROFFQ and the weighed food records (Pearson’s correlation coefficients and Bland–Altman plots) and quintile rankings to measure group level agreement. The adequate correlations observed between questionnaire responses suggest that the AD-CSIROFFQ is reliable. All nutrient intakes were acceptable for ranking of individuals on a group level, whilst the agreement levels with respect to the weighed food records for 11 of the 46 nutrients show validity in terms of their individual level absolute intake. The AD-CSIROFFQ makes an important contribution to the tools available for assessing usual dietary intake in groups of older adults with respect to AD research.

## 1. Introduction

Accurate assessment of usual dietary intake is crucial to understanding the association between diet and chronic diseases; incorrect information may lead to false associations between dietary factors and disease risk and prevention. There is a need to develop and validate methods of assessing food and beverage intake to facilitate the development of guidelines for the prevention of diet-related chronic diseases.

Food frequency questionnaires (FFQs) are a relatively inexpensive method of measuring usual food intake over an extended period, particularly among large cohorts. FFQs are easy to administer with low participant burden and are therefore a more practical method to collect dietary data for large-scale studies, compared with 24-h recalls or food records which may require trained interviewers. However, dietary questionnaires need to be both specific to the population being analysed, and to the disease of interest; currently, there is no Alzheimer’s disease (AD)-specific FFQ available.

Accuracy and precision in measuring intake of individual dietary constituents are important issues in the analysis and evaluation of results from studies on the associations between diet and chronic diseases. The gold standard and most common method of validating a dietary questionnaire is through comparison of responses from the FFQ to actual food intakes documented for a representative number of days using weighed food records [1]. The reliability or precision of the tool can be estimated by administering the FFQ at two time points to the same group of people and assessing the level of agreement between the two responses [2].

The Commonwealth Scientific and Industrial Research Organisation (CSIRO) FFQ is a quantitative questionnaire containing over 200 foods and beverages in the following categories: cereals and breads, dairy and eggs, meats and mixed dishes, chicken, fish and seafood, takeaway food, vegetables, fruit, beverages, and snacks and sweets; a paper version of the CSIROFFQ has been validated for use in the Australian population [3,4,5,6]. For the current study, a computerised version of the original FFQ was modified to include questions on foods of interest in the field of AD research with the resultant questionnaire termed the AD-CSIROFFQ. These new questions were selected based on current literature describing major dietary contributors associated with AD risk [7,8,9,10,11]. Questions were added to capture information in relation to dietary intake of chocolate type, oils (e.g., grape seed, coconut oil), wine type, green and white tea, pomegranates and pomegranate juice, types of grapes, types of berries, offal (e.g., kidney, liver, heart), types of meat used in stew, casserole, curry and goulash, types of rice, types of nuts and seeds, nutritional supplements, and herbs and spices.

The aim of this study was to determine the suitability of the AD-CSIROFFQ for use in studies of AD and ageing through assessment of (1) reliability of the AD-CSIROFFQ by comparing the responses of cognitively normal older adults when the questionnaire was administered on two occasions and (2) validity of the AD-CSIROFFQ relative to four-day weighed food records.

## 2. Materials and Methods

### 2.1. Participants

This study was undertaken by 148 older adults drawn from three cohorts in Perth, Western Australia: 88 participants from the Australian Imaging, Biomarkers and Lifestyle Study of ageing [12], 51 participants from the Western Australian Memory Study [13,14,15,16], and 9 participants from the Western Australian Participant Pool (a register of potential participants for studies to recruit from). All participants were classified as cognitively normal at the time they completed the FFQ validation study, as determined by a Mini-Mental State Examination (MMSE) score of 25 or higher.

### 2.2. Study Design

**Figure 1:**
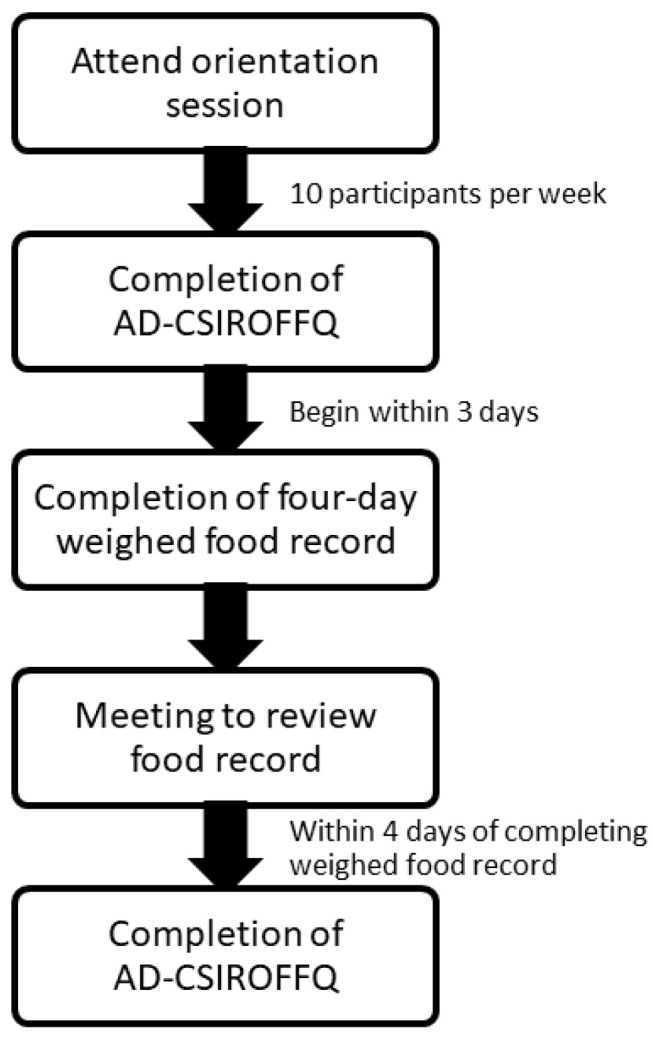
Schematic of the study design. AD-CSIROFFQ, (Alzheimer’s disease-Commonwealth Scientific and Industrial Research Organisation Food Frequency Questionnaire).

Participants completed an initial computerised AD-CSIROFFQ followed by a consecutive four-day weighed food record (started within the next three days) and then a repeat computerised AD-CSIROFFQ (Figure 1).

### 2.3. Alzheimer’s Disease Food Frequency Questionnaire

The AD-CSIROFFQ is an online FFQ with over 280 food and beverage items which includes serving sizes that can be altered by the respondent. The FFQ also contains options for each item related to food preparation and cooking techniques, as well as options for selection of specific type of the item (e.g., type of milk used, type of bread consumed, type of fats used as spread or in cooking, type of fish consumed, etc.), which yields a plethora of final options of the 280 initial food and beverage items. To minimise fatigue, the FFQ was designed to have the foods arranged into categories and automatically saves data entered, allowing participants to leave the questionnaire and return later to complete it in multiple sittings. Participants cannot move to the next section until all questions are fully answered in the current section, ensuring the questionnaire is complete. The AD-CSIROFFQ takes approximately 45 min to complete, requires access to the Internet, and assesses usual daily intake over the preceding 12 months. Grams per day intake of foods and beverages is provided following completion of the questionnaire. This intake was then analysed using FoodWorks Professional version 7.0 (Xyris Software Pty Ltd., Brisbane, Queensland, Australia.) and the AUSNUT 2007 database of Australian Foods to yield grams per day of nutrients consumed.

### 2.4. Four-Day Weighed Food Record

Participants attended an orientation session, during which detailed verbal and written instructions regarding the completion of the four-day weighed food records were given by an Accredited Practising Dietitian. Digital electronic kitchen scales (model 1348, Propert) weighing to the nearest gram and modular measuring cup and spoon sets were provided to each participant, as was a sample of a completed food record. Each participant was requested to record the weight of all food and beverages consumed during the four-day period from Sunday through Wednesday (at home or away from home) and to record brand names, methods of food preparation, and ingredients of recipes in a specially designed diary. In addition, participants were asked to record the amount of any leftover food at the end of a meal or snack, either at home or away from home, and to report any dietary supplements consumed. All food records were reviewed face-to-face with the participant upon completion of the four-day period to clarify details or missing information. Four-day weighed food records were analysed using FoodWorks Professional version 7.0 and the AUSNUT 2007 database of Australian Foods to yield grams per day of nutrients consumed.

### 2.5. Statistical Analysis

Statistical analyses were performed using the IBM Statistical Package for the Social Sciences, Version 25.0 (SPSS Inc., Chicago, IL, USA). A *p*-value of <0.05 determined a significant result. Mean values, standard deviations (SD), and percentages are provided for the demographics for the reliability and validation study cohort.

#### 2.5.1. Reliability Study

The raw data for the majority of food items was not normally distributed; therefore, Spearman’s rank correlation coefficients were used to compare the correlation between food and beverage intakes from the first and second administration of the AD-CSIROFFQ.

#### 2.5.2. Validation Study

The raw data for the majority of nutrients were not normally distributed; logarithmic transformation produced normalised data that maximised the Kolmogorov–Smirnov test statistic. Following log transformation, the nutrient data were energy-adjusted using linear regressions. Raw data were used to calculate mean and SD intakes for the 46 nutrients analysed. Bivariate Pearson’s correlation coefficients were calculated for the log-transformed and energy-adjusted nutrient data to estimate the associations between the two methods, i.e., the AD-CSIROFFQ and the weighed food record.

Agreement between both methods was assessed using Bland–Altman plots [17], which represent the individual differences between the two measurements graphed against the mean of the measurements. Interpretation of the Bland–Altman results was based on three categories: “good agreement”, “fairly good agreement”, and “poor agreement”, depending on whether the difference between the two measurements is approximately equal to 1, 2, or 3 standard deviations of the average nutrient intake, respectively.

The agreement between methods of the relative rankings of nutrient intakes was assessed by classifying subjects into quintiles and cross-tabulating, using the log-transformed and energy-adjusted data. Weighted kappa statistics (κ) were calculated to measure the strength of agreement with 0.61–0.80 being “substantial”, 0.41–0.60 “moderate”, 0.21–0.40 “fair”, and less than 0.20 “slight” [18].

## 3. Results

Of the cohort of 148 participants, 136 completed the two administrations of the AD-CSIROFFQ and were included in the reliability study analysis. This cohort included 44 males (32%) and had a mean and SD age of 73.19 ± 6.28 years. All 148 individuals participated in the validation study; of these, 48 were male (32%) and the mean age was 73.20 ± 6.43 years.

### 3.1. Reliability Study

To investigate the reliability of the AD-CSIROFFQ, we assessed the correlation of intakes of 508 food and beverage items from two administrations of the questionnaire (Table 1). Of the 508 items, 182 were highly correlated (*p* < 0.001), 27 were moderately correlated (*p* < 0.01), and 34 were weakly correlated (*p* < 0.05). Sixty-six items were not significantly correlated; these items comprised mostly individual fruits and vegetables, and takeaway foods for which low consumption levels were reported in both administrations of the questionnaire. Spearman’s rank correlation coefficients ranged from −0.866 for subs/wraps with seafood as the main filling (three participants reporting consumption) to 1.000 for packet soup—mushroom. One hundred and ninety-nine items could not be analysed due to their low reporting rate; 19 of these items were from the AD-related questions added to the FFQ, and included intakes of chicken and beef liver, beef and veal kidney, lamb heart, and palm oil.

Intake of herbs and spices reported following completion of the AD-CSIROFFQ on two occasions was compared using binary scores assigned to “yes” or “no” answers to questions relating to whether an individual had consumed each of 19 herbs or spices in the last fortnight. Out of a possible 2584 responses, 2083 (80.61%) matched between the two administrations. The herbs and spices most commonly inconsistent in their responses were ginger, parsley, and rosemary. Only 19 of the 136 participants had all 19 answers identical.

The intake of a total of 964 dietary supplements was reported overall; 288 were reported in one FFQ administration and not the other, whilst 338 were reported in both administrations. Of the 288 reported in only one administration, 235 had a consumption frequency of “yearly” or “rarely”. Of the 338 supplements reported in both administrations, 50 had inconsistent consumption frequency stated, 120 had different brands of supplements stated, and 22 had different supplement strength reported (only fish oil and krill oil had supplement strength requested). The final question concerning supplement intake was an open-ended question regarding intake of any other supplements not listed previously within the questionnaire (e.g., magnesium, iron, and probiotics): 37 participants stated that they consumed an additional supplement in both administrations of the questionnaire; 27 participants stated that they consumed an additional supplement in one administration of the questionnaire only.

### 3.2. Validation Study

Bivariate Pearson’s correlation coefficients estimating the association of the nutrient intakes between the AD-CSIROFFQ and weighed food records ranged from −0.092 (vitamin B6) to 0.570 (alcohol). Of the 46 nutrients analysed, 41 were significantly positively correlated, with 34 at the *p* < 0.001 level, 5 at the *p* < 0.01 level, and 2 at the *p* < 0.05 level (Table 2). Nutrients that were not correlated were vitamin B6, vitamin B12, sodium, zinc, and energy.

**Figure 2:**
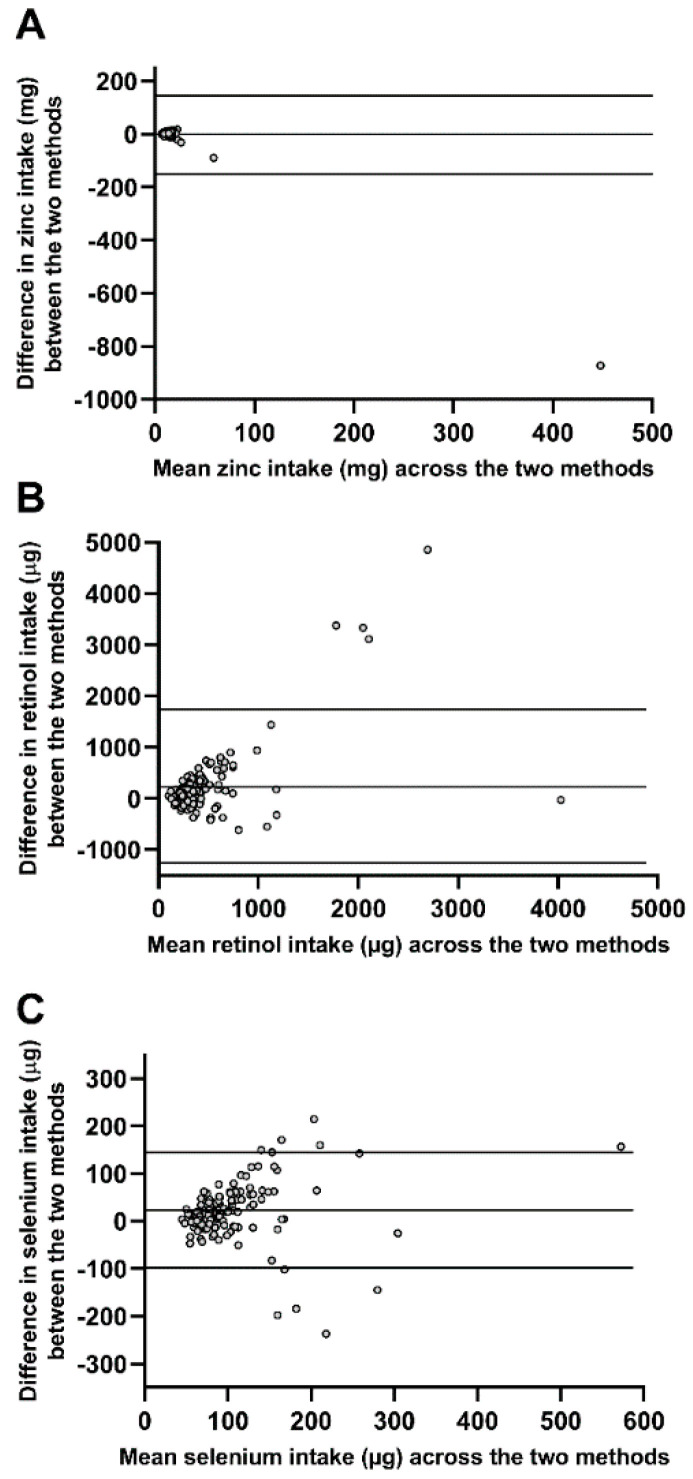
Bland–Altman plots depicting examples of “good agreement” (**A**), “fairly good agreement” (**B**), and “poor agreement” (**C**) of nutrient intakes determined using the AD-CSIROFFQ and four-day weighed food records. The difference in intake of nutrients between the two methods is plotted against the mean intake across the two methods for each individual. Upper horizontal line shows one standard deviation above the mean intake, middle horizontal line is the mean intake, and lower horizontal line shows one standard deviation below the mean intake. Abbreviations: AD-CSIROFFQ, Alzheimer’s disease-Commonwealth Scientific and Industrial Research Organisation Food Frequency Questionnaire.

Agreement between both methods with respect to nutrient intake was assessed using Bland–Altman plots (“Level of Agreement”; Table 2). Six of 46 nutrients showed “good agreement”, 29 nutrients showed “fairly good agreement”, and 11 nutrients showed “poor agreement”. Example plots depicting “good”, “fairly good”, and “poor agreement” are shown in Figure 2.

Quintiles of nutrient intake levels were also compared between the AD-CSIROFFQ and the weighed food records. The ranking analysis indicated that between 54% and 88% of subjects (mean 63%) were classified into either the same or adjacent nutrient quintiles in both methods (% in same quintile + % in adjacent quintile), with the highest percentages observed for niacin, docosahexaenoic acid, eicosapentaenoic acid, and alcohol. The two methods grossly misclassified (disagreement by three or four quintiles) between 3% and 22% of subjects (average 15%), with the highest misclassification seen for water, carbohydrate, and energy. The strength of quintile agreement between the two methods was “substantial” for 4 nutrients, “moderate” for beta carotene intake, “fair” for 7 nutrients, and “slight” for the remaining 34 nutrients (Table 3).

## 4. Discussion

The reliability and comparative validity of the customised online AD-CSIROFFQ were assessed by comparing actual food intake data from the AD-CSIROFFQ at two administrations (reliability), and by comparing nutrient intake documented using four-day weighed food records with data generated from the first administration of the AD-CSIROFFQ (validity).

Of the 309 food and beverage items that had sufficient consumption levels for analysis of reliability, over 78% were significantly correlated between questionnaire administrations. The items lacking significant correlation were mainly takeaway foods and individual fruit and vegetable items that were consumed at low levels. These results indicate that the overall repeatability, and thus reliability, of the AD-CSIROFFQ is good.

Validity of an FFQ is the degree to which the instrument measures the diet of the subjects it was designed to study. To determine validity, we used two methods to assess agreement of absolute nutrient intake between the AD-CSIROFFQ and four-day weighed food records. First, Pearson’s correlation coefficients showed that 41 of the 46 nutrients analysed were significantly positively correlated, with 34 of these highly correlated, providing evidence that intake levels in the AD-CSIROFFQ are associated with intakes documented via the weighed food records. Second, Bland–Altman plots assessing individual agreement of nutrient intakes between the two methods indicated that 35 of the 46 nutrients showed “good” or “fairly good” agreement.

We also examined the ranking of individuals into quintiles of nutrient intake and compared quintiles between the AD-CSIROFFQ and weighed food records. The AD-CSIROFFQ demonstrated an acceptable ability to rank subjects into the same or adjacent quintile as the weighed food records. On average, for all nutrients, 63% of participants were classified into the same or adjacent nutrient intake quintile, with the highest observed agreement of 88% for alcohol intake. On average, only 15% of participants were grossly misclassified by three or four quintiles, with the lowest value of 3% again for alcohol intake. Our quintile agreement results are comparable to those reported in a previous validation study using the paper-based CSIROFFQ in 62 women aged 31–60 years, where approximately 70% of reported intakes were in the same or adjacent quintile [6]. Yet, our group level agreement results are lower than values reported in a validation study of 14-year-old adolescents (*n* = 785), where the percentage agreement was 80%–90%. Notably, however, the adolescent study utilised a three-day weighed food record as the reference tool, and data were assigned to tertiles rather than quintiles [3]; these methodological differences increased the likelihood of group level agreement in the adolescent study.

In the present study, due to the quintile agreement being similar to that reported in other studies [3,6], we conclude that the AD-CSIROFFQ is a suitable tool for determining high or low intakes of nutrients, thereby yielding valuable information at the group level. For epidemiological purposes, ranking of individuals may be more relevant than determining absolute intakes. FFQs are commonly used in studies to examine the association between diet and chronic diseases [2], and for these studies, the ability of the FFQ to rank individuals on usual nutrient intakes may be more important than the estimation of absolute intake. For example, dietary patterns utilise individuals’ intake rankings to investigate the association between diet and chronic diseases; dietary patterns frequently use median intake values to assign individuals to a binary value for inclusion into a dietary pattern score.

Overall, the evidence from the current study, particularly from agreement levels, leads to the recommendation that the AD-CSIROFFQ should be used selectively if measuring absolute nutrient intakes at the individual level. Specifically, our results provide evidence that the AD-CSIROFFQ measures VLC N3, alpha linolenic acid, docosahexaenoic acid, and caffeine accurately, based on good agreement and significant correlations, and a further 27 nutrients reasonably accurately, based on fairly good agreement and significant correlations; therefore, the AD-CSIROFFQ is a useful tool in this respect. Available carbohydrate, sugars, and dietary fibre are important nutrients for disease research, and whilst there was high correlation (*p* < 0.001) of intakes between the AD-CSIROFFQ and weighed food records, poor agreement was observed in the Bland–Altman plots, suggesting that one should proceed with caution in utilisation of the AD-CSIROFFQ for measurement of absolute intake of these nutrients.

Weighed food records are acceptable gold standards because they are thought to provide more accurate and representative food intake data within the period covered by the FFQ. It is important that the errors of both methods are independent to avoid spuriously high estimates of validity. Among the available and feasible comparison methods for validating an FFQ, diet records are likely to have the least correlated errors. The main source of error in the reference method is the possibility of a change in diet due to the measurement process itself, despite requests that participants consume their normal diet, and reassurance from all that this was the case. Conversely, the main sources of error in the questionnaire are restrictions imposed by a fixed list of foods, memory of foods consumed, interpretation of questions, and the assumption of average serving sizes for most foods. By contrast, diet records are collected prospectively and do not depend on memory. They allow a more direct assessment of portion sizes, and errors in interpretation relate to the dietitian coding the records rather than to the subjects. Agreement between the two methods of dietary data collection may be affected by reporting of items in the FFQ which were not consumed during the four-day weighed food record or were consumed by few people (e.g., specific types of fish and breakfast cereals). The day of the week may also influence the result of validation studies. In our study, the greatest proportion of records was collected on weekdays (75%) with 25% collected on a weekend day; this division was deliberate, as it is perceived that usual dietary intake occurs during the week with greater variation observed at weekends [1,2].

Irrespective of the limitations described above, the present study provides evidence of acceptable reliability and validity of the AD-CSIROFFQ as a measure of dietary intake, particularly in the AD field for Australian cohorts. The adequate correlations observed between test–retest responses suggest that the AD-CSIROFFQ is reliable. The agreement levels with respect to the weighed food records for a number of nutrients shows validity in terms of their individual intake, with all intakes being acceptable for ranking of individuals on a group level, and thus providing an acceptable assessment of long-term dietary intake in older adults. In summary, the AD-CSIROFFQ makes an important contribution to the tools available for assessing usual dietary intake in older adults with respect to AD research.

## Figures and Tables

**Table 1:** Associations between reported food and beverage item intake from two administrations of the Alzheimer’s disease-CSIRO Food Frequency Questionnaire.

Food or Beverage Item	Spearman’s Rank Correlation Coefficient	Number of Participants Consuming Item	Food or Beverage Item	Spearman’s Rank Correlation Coefficient	Number of Participants Consuming Item	Food or Beverage Item	Spearman’s Rank Correlation Coefficient	Number of Participants Consuming Item
Vegetables			Meat and Mixed Dishes			Dairy and Eggs		
Avocado—fresh	0.810 ***	105	Roast lamb	0.558 ***	76	Cheese—regular variety	0.688 ***	95
Baked beans—canned	0.730 ***	92	Rissole/meat patty	0.600 ***	63	Cheese—fat reduced variety	0.830 ***	19
Bean sprouts	0.338	19	Asian stir-fry	0.602 ***	78	Cottage cheese	0.674 ***	33
Beetroot—canned	0.813 ***	81	Mincemeat as a sauce	0.632 ***	78	Ricotta cheese	0.493 **	27
Bok choy	0.610 ***	47	Ham	0.650 ***	90	Soft cheese	0.585 ***	68
Broccoli	0.623 ***	124	Beef stew/casserole	0.667 ***	49	Milk—whole milk	0.776 ***	35
Brussels sprouts	0.794 ***	68	Pizza	0.684 ***	24	Milk—reduced fat milk	0.606 ***	38
Cabbage	0.675 ***	90	Steak	0.689 ***	105	Milk—reduced fat milk + added calcium and nutrients	0.400	4
Capsicum	0.658 ***	122	Roast beef/veal	0.687 ***	64	Milk—skim/non-fat milk	0.534 *	20
Carrots	0.526 ***	131	Gravy—powder prepared with water	0.682 ***	42	Glass of milk	0.686 ***	38
Cauliflower	0.722 ***	115	Pate/liver paste	0.640 ***	34	Ice cream—regular	0.757 ***	59
Celery	0.625 ***	109	Gravy—prepared from pan drippings	0.641 ***	28	Ice cream—fat reduced	0.748 *	10
Coleslaw	0.722***	96	Homemade soup—vegetable	0.699 ***	34	Milkshake/thickshake—regular fat milk	0.732 **	15
Cucumber	0.717 ***	123	Crumbed veal	0.677 **	20	Flavoured milk—regular fat	0.956 **	6
Fresh fruit salad	0.552 ***	71	Lamb chop or cutlet	0.726 ***	85	Flavoured milk—reduced fat	0.900 *	5
Fried mixed vegetables	0.427 **	47	Bacon	0.734 ***	93	Butter	0.645 ***	88
Garlic	0.703 ***	102	Pork chop	0.749 ***	64	Margarine	0.638 ***	60
Gherkins/pickled onions	0.737***	52	Roast pork/pork fillet	0.751 ***	57	Cream—regular thickened (35%)	0.848 ***	36
Green beans	0.569 ***	113	Beef curry	0.758 ***	19	Cream—light thickened (18%)	0.500	3
Green peas	0.570 ***	117	Meat pie	0.785 ***	25	Dairy-style dessert	0.291	8
Green peas—canned	0.500	3	Sausages	0.797 ***	90	Ice block/icy pole	0.494	8
Lentils—dried/canned	0.564 ***	50	Chicken stew/casserole	0.790 ***	15	Custard—regular vanilla flavoured	0.614 ***	36
Lettuce	0.447 ***	124	Continental sausage	0.820 ***	25	Yoghurt—Greek-style natural	0.902 ***	32
Mushrooms—fresh	0.579 ***	75	Homemade soup—pea and ham	0.979 ***	9	Yoghurt—flavoured regular fat (3%)	0.900 *	5
Mushrooms—fried	0.476 ***	79	Packet soup—mushroom	1.000 ***	2	Yoghurt—flavoured low fat (.5%)	0.949	4
Olives	0.693 ***	85	Frankfurters/saveloys	0.943	4	Yoghurt—natural regular fat (4%)	0.614	8
Onion—fried	0.559 ***	49	Lamb liver	0.715 *	8	Yoghurt—natural low fat (.5%)	0.564	5
Onion—raw/baked/boiled	0.560 ***	79	Beef goulash	0.609 *	11	Yoghurt—natural reduced fat (2%)	0.882 **	7
Other beans—dried or canned	0.650 ***	57	Lamb curry	0.615	9	Boiled/poached egg	0.555 ***	95
Other pickled vegetables	0.696 **	15	Lamb stew/casserole	0.716	6	Fried egg	0.633 ***	61
Pumpkin	0.705 ***	118	Canned soup—tomato	0.591	8	Omelette/scrambled eggs	0.526 ***	92
Silver beet/spinach	0.594 ***	90	Canned soup—meat and vegetable	0.975 **	5	**Cereals and Cereal Foods**		
Strawberries	0.667 ***	121	Homemade soup—chicken and vegetable	0.643 *	14	Bread rolls	0.504 ***	67
Sweet potato	0.614 ***	111	Mornay dishes	0.483 ***	49	Flat breads	0.514 **	32
Sweetcorn	0.738 ***	88	Chicken curry	0.782 ***	30	Crumpets or English muffins	0.664 ***	31
Sweetcorn—canned	0.548 ***	38	Vegetarian curry/stew casserole	0.342 *	43	Croissants	0.807 ***	19
Tomato—fresh	0.536 ***	133	Homemade soup—pumpkin	0.641 *	11	Crispbread/rice cake/cracker	0.606 ***	67
Tomato—fried/grilled	0.523 ***	77	Canned soup—pea and ham	0.667	5	Muesli bar/health bar	0.852 ***	43
Tomatoes—canned	0.552 ***	88	Packet soup—vegetable	−0.866	3	Fruit loaf/currant bread	0.801 ***	40
Turnip, swede	0.605 **	23	Mincemeat dish	0.082	69	Cereal-based sweet desserts	0.682 ***	22
Vegetarian stir-fry	0.570 ***	45	Packet soup—chicken noodle	0.300	5	Barley cooked	0.858 ***	16
Zucchini	0.605 ***	81	Homemade soup—meat and vegetable	−0.168	10	Porridge/oatmeal—unsweetened	0.864 ***	57
Potato—roasted	0.427 ***	96	Canned soup—mushroom	−0.500	3	Plain bran	0.886 ***	12
Jacket potatoes	0.475 ***	66	Lamb kidney	−0.094	6	Muesli—packaged untoasted/natural	0.521 **	28
Potato—mashed with milk	0.708 ***	94	Luncheon meat/devon	0.105	4	Muesli—homemade	0.562 **	28
Potato salad	0.790 ***	78	Parmagiana	0.455	8	Muesli—packaged toasted	0.588	6
Oven baked hot chips prepared at home	0.615 ***	35	Savoury pies/pastries	0.434 **	39	LSA mixture	0.718 **	15
Potato—boiled	0.645 ***	85	**Chicken, Fish, and Seafoods**			Sanitarium Weet-Bix	0.623 *	14
**Fruit**			Canned salmon—red salmon	0.422	10	Instant noodles	0.635 **	16
Apple, pear—fresh baked	0.763 ***	121	Canned salmon—pink salmon	0.693 ***	29	Asian noodles	0.643 ***	34
Apricot—fresh	0.614 ***	66	Canned salmon—Australian salmon	0.310	7	Couscous	0.589 ***	33
Banana	0.713 ***	128	Canned tuna—regular variety canned in spring water	0.577 **	27	Plain pasta	0.573 ***	83
Blackberries	0.604 *	16	Canned tuna—regular variety canned in oil	0.776 **	13	Filled pasta	0.538 *	21
Blueberries	0.682 ***	58	Canned tuna—regular variety canned in brine	0.000	4	Packet pasta and sauce	0.479 *	23
Cherries	0.547 ***	72	Canned tuna—flavoured variety canned in spring water	0.755	7	Brown rice	0.733 **	18
Cranberries	0.422 *	29	Canned tuna—flavoured variety canned in oil	0.632	4	Fried rice	0.614 ***	41
Figs—fresh	0.658 ***	42	Other canned fish	0.543 ***	44	White rice	0.637 ***	91
Fruits canned in juice	0.684 ***	40	Steamed/grilled/boiled fish—other inexpensive fish—no coating	0.353	6	Risotto	0.849 ***	31
Fruits canned in syrup	0.475	9	Steamed/grilled/boiled fish—barramundi—no coating	0.679	8	**Sweets and Snacks**		
Fruits canned in water	0.441	10	Steamed/grilled/boiled fish—salmon—no coating	0.776 ***	23	Chocolate-covered bar	0.592 ***	37
Green grapes—fresh	0.521 ***	106	Steamed/grilled/boiled fish—snapper—no coating	0.436	10	Dark chocolate	0.657 ***	90
Kiwifruit	0.695 ***	72	Seafood	0.491 ***	69	Milk chocolate	0.675 ***	62
Mango	0.607 ***	98	Fried fish—salmon—no coating	0.949	4	White chocolate	0.454	15
Melon	0.469 ***	90	Fried fish—snapper —flour	0.500	3	Vegemite, marmite, etc.	0.714 ***	71
Nectarine—fresh	0.507 ***	105	Oven-baked fish—salmon—no coating	0.376	7	Honey, jam, marmalade	0.603 ***	89
Orange, mandarin, grapefruit	0.726 ***	111	Oven-baked fish—Snapper—no coating	−0.500	3	Peanut paste	0.763 ***	57
Other berries	0.540 *	15	Crumbed chicken	0.522 **	28	Fancy sweet biscuits	0.463 **	52
Other dried fruit	0.532 ***	53	Chicken breasts, thighs, or wings—without skin	0.621 ***	83	Salted biscuits	0.618 ***	71
Pawpaw	0.668 **	17	Chicken breasts, thighs, or wings—with skin	0.474 *	19	Plain sweet biscuits	0.648 ***	64
Peach—fresh	0.477 ***	86	Roast/barbeque chicken	0.240	42	Cake/sweet muffin	0.654 ***	90
Pineapple—fresh	0.559 ***	40	Chicken cooked in simmer sauce	−0.002	16	Potato crisps/Twisties/corn chips, etc.	0.655 ***	48
Plum—fresh	0.529 ***	91	**Takeaway Foods**			Lollies, toffees	0.599 **	21
Pomegranates	0.693	8	Garlic bread	0.481 ***	50	Sweet bun/doughnut	0.487 *	27
Raisins, sultanas, or currants	0.752 ***	83	Fries	0.590 ***	38	Fruit pie or pastry or fritters	0.208	24
Raspberries	0.424 *	25	Hot chips	0.647 ***	52	Popcorn—commercial	0.866	3
Red/black grapes—fresh	0.475 ***	102	Pizza	0.632 ***	64	Sugar/honey added to cereals	0.745 ***	28
Watermelon	0.505 ***	92	Meat/chicken pie	0.055	11	Sugar/honey added to hot beverages	0.763 ***	28
**Beverages**			Pastie	0.640 ***	30	Chia seeds	0.904 ***	18
Milo/Quik/Ovaltine/cocoa/hot chocolate	0.609 ***	45	Hamburgers—large	0.601 **	18	Flax seeds	0.866	3
Green tea	0.701 ***	62	Hamburgers—small	0.785 **	11	Pumpkin seeds	0.767 ***	25
Herbal tea	0.746 ***	56	Sausage roll	0.707 ***	34	Pumpkin seeds (roasted)	0.416	9
Black tea	0.878 ***	76	Deep fried seafood	0.221	19	Sesame seeds	0.796 ***	20
White tea	0.500	4	Deep fried chicken	0.690 **	14	Sesame seeds (roasted)	0.679	7
Coffee	0.880 ***	118	Deep fried battered fish	0.763 ***	48	Sunflower seeds	0.601 **	28
Water/spring water	0.758 ***	99	Potato cakes/fritters	0.195	8	Almonds	0.821 ***	61
Unflavoured mineral water	0.813 ***	20	Spring/chiko roll	0.424	10	Almonds (roasted)	0.875 ***	20
Pure fruit juice	0.828 ***	44	Fried dim sum	0.771	6	Brazil nuts	0.852 ***	29
Fruit drink	0.932 ***	12	Steamed dim sum	0.366	10	Cashew nuts	0.389 *	27
Pomegranate juice	0.500	3	Chicken sushi rolls	0.598 ***	31	Cashew nuts (roasted)	0.625 ***	30
Vegetable juice	0.644 **	16	Meat sushi rolls	0.654*	13	Hazelnuts	0.495	7
Diet fizzy drink	0.676 **	18	Seafood sushi rolls	0.553 ***	49	Hazelnuts (roasted)	0.638	6
Regular fizzy drink	0.523 *	23	Vegetarian sushi rolls	0.688 ***	31	Macadamia nuts	0.841 ***	15
Low-calorie cordial	0.882 *	6	Wraps/subs with chicken or fish as the main filling	0.538**	23	Macadamia nuts (roasted)	0.806 **	11
Regular cordial	0.626 *	13	Wraps/Subs with beef, lamb, or pork as the main filling	0.338	13	Other nuts	0.278	7
Red wine	0.810 ***	64	Wraps/subs with sandwich meat as the main filling	0.811	5	Peanuts (salted or unsalted, roasted)	0.783 ***	30
Sparkling wine or Champagne	0.861 ***	48	Subs/wraps with seafood as the main filling	−0.866	3	Pecans	0.877 ***	12
Rose wine	0.719 *	10	**Miscellaneous**			Pine nuts	0.505	10
Medium white wine	0.878 ***	69	Canola oil	0.634 **	20	Pine nuts (roasted)	0.857 **	8
Sherry/port/liqueur	0.874 ***	22	Coconut oil	0.733 **	13	Pistachios	0.737 *	10
Spirits	0.917 ***	28	Dripping/lard	0.000	5	Pistachios (roasted)	0.922 ***	12
Low-alcohol beer	0.862 ***	28	Extra virgin olive oil	0.683 ***	109	Walnuts	0.628 ***	53
Regular beer	0.937 ***	29	Olive oil	0.514 ***	43	Walnuts (roasted)	0.060	7
			Peanut oil	−0.308	7			
			Sunflower oil	0.405 *	12			
			Marinades and other thick sauces	0.519 ***	47			
			Thick sauces	0.622 ***	62			
			Mayonnaise—regular	0.765 ***	39			
			Mayonnaise—fat reduced	0.563 **	24			
			Salad dressing—full fat oil and vinegar style	0.454 *	30			
			Salad dressing—full fat creamy style	−0.400	4			
			Salad dressing—reduced/fat free vinegar style	0.716 ***	22			

Bold indicates significance (* *p* < 0.05, ** *p* < 0.01, *** *p* < 0.001); *n* = 136. Abbreviations: CSIRO, Commonwealth Scientific and Industrial Research Organisation; LSA, linseeds, sunflower seeds and almonds.

**Table 2:** Associations between energy and nutrient intakes obtained from the Alzheimer’s disease-CSIRO Food Frequency Questionnaire and four-day weighed food records.

Nutrient	Mean WFR ^a^	SD WFR ^a^	Mean FFQ ^a^	SD FFQ ^a^	Pearson’s Correlation Coefficient ^b^	Level of Agreement ^b,c^
Energy	8049.0	1938.0	11,391.6	3844.9	0.160	Fairly Good
Protein	84.4	20.2	113.0	36.2	0.336 ***	Fairly Good
Total fat	76.6	25.0	129.7	58.3	0.356 ***	Fairly Good
Saturated fat	27.4	10.7	44.0	21.4	0.438 ***	Fairly Good
Trans fatty acids	1.2	0.6	2.1	1.2	0.418 ***	Poor
Polyunsaturated fat	13.3	5.9	20.1	10.2	0.403 ***	Fairly Good
Monounsaturated fat	29.0	11.5	54.7	26.9	0.285 ***	Fairly Good
Cholesterol	274.7	121.4	351.0	152.6	0.256 **	Poor
Available carbohydrate	192.8	61.7	236.2	89.7	0.465 ***	Poor
Sugars	96.9	36.3	134.9	56.2	0.441 ***	Poor
Starch	94.8	38.5	99.9	48.4	0.428 ***	Fairly Good
Dietary fibre	26.6	7.2	42.3	14.5	0.343 ***	Poor
Thiamin	3.2	10.2	2.4	2.1	0.165 *	Fairly Good
Riboflavin	2.5	3.1	2.7	1.1	0.335 ***	Fairly Good
Niacin	23.4	18.6	31.4	10.8	0.266 **	Fairly Good
Niacin equivalents	40.3	20.1	52.9	17.3	0.254 **	Fairly Good
Vitamin C	150.3	200.6	249.2	117.3	0.280 **	Fairly Good
Vitamin E	15.3	15.3	23.2	11.5	0.386 ***	Fairly Good
Alpha tocopherol	11.5	7.8	19.9	8.9	0.456 ***	Fairly Good
Vitamin B6	5.2	15.7	2.4	0.8	−0.092	Poor
Vitamin B12	7104.8	84214.4	6.6	3.6	−0.032	Good
Total folate	535.4	359.3	585.1	219.2	0.360 ***	Fairly Good
Folic acid	168.8	180.1	81.9	99.5	0.357 ***	Fairly Good
Dietary folate equivalents	645.7	437.4	639.9	257.1	0.405 ***	Fairly Good
Vitamin A	1172.8	1394.3	2412.7	1391.4	0.317 ***	Fairly Good
Retinol	334.9	374.4	606.2	833.6	0.340 ***	Fairly Good
Beta carotene equivalents	4263.2	2724.1	10,841.8	6524.6	0.426 ***	Fairly Good
Beta carotene	3500.5	2319.5	8564.7	5258.0	0.430 ***	Fairly Good
Sodium	2079.2	859.1	2448.4	1042.4	0.138	Fairly Good
Potassium	3244.5	751.9	5241.3	1731.7	0.390 ***	Poor
Magnesium	397.1	159.9	485.6	168.0	0.384 ***	Poor
Calcium	1020.7	447.2	1146.7	489.8	0.377 ***	Fairly Good
Phosphorus	1488.8	337.2	2006.2	636.9	0.413 ***	Fairly Good
Iron	11.7	3.9	15.8	5.4	0.328 ***	Fairly Good
Zinc	17.7	72.5	14.2	4.5	−0.044	Good
Selenium	94.9	61.9	114.9	69.7	0.561 ***	Poor
Iodine	173.0	77.0	165.3	77.3	0.391 ***	Fairly Good
VLC N3	16.8	106.8	0.8	0.8	0.462 ***	Good
Linoleic Acid	11.8	12.6	16.5	9.1	0.372 ***	Fairly Good
Alpha Linolenic Acid	33.5	265.6	2.3	1.5	0.192 *	Good
Eicosapentaenoic Acid	5.4	60.6	0.3	0.3	0.363 ***	Fairly Good
Docosapentaenoic Acid	0.1	0.1	0.2	0.1	0.436 **	Poor
Docosahexaenoic Acid	3.7	40.4	0.3	0.3	0.455 **	Good
Caffeine	209.1	502.3	137.2	130.3	0.474 **	Good
Water	3380.5	6702.8	3119.3	2283.5	0.279 **	Fairly Good
Alcohol	10.2	13.6	10.6	15.0	0.570 ***	Poor

Bold indicates statistical significance (* *p* < 0.05, ** *p* < 0.01, *** *p* < 0.001); *n* = 148. ^a^ Uses raw data. ^b^ Uses log-transformed and energy-adjusted data. ^c^ “Good agreement”: the difference between the two methods is approximately equal to one standard deviation of the average nutrient intake. “Fairly good agreement”: the difference between the two methods is approximately equal to two standard deviations of the average nutrient intake. “Poor agreement”: the difference between the two methods is approximately equal to three standard deviations of the average nutrient intake. Abbreviations: CSIRO, Commonwealth Scientific and Industrial Research Organisation; FFQ, food frequency questionnaire; SD, standard deviation; VLC N3, very long chain n-3 polyunsaturated fatty acids; WFR, weighed food record.

**Table 3:** Quintile percentage agreement between energy and nutrient intakes determined from the Alzheimer’s disease-CSIRO Food Frequency Questionnaire and four-day weighed food records.

Nutrient	Same Quintile, %	Adjacent Quintile, %	Gross Misclassification, % ^a^	Weighted κ ^b^	Strength of Agreement ^c^
Energy	23.81	34.01	22.45	0.249	Fair
Protein	25.85	31.97	16.33	0.076	Slight
Total fat	23.81	40.14	17.01	0.249	Fair
Saturated fat	25.85	34.69	14.97	0.076	Slight
Trans fatty acids	30.50	36.17	16.31	0.002	Slight
Polyunsaturated fat	29.93	36.73	15.65	0.003	Slight
Monounsaturated fat	25.17	34.01	16.33	0.117	Slight
Cholesterol	29.25	36.05	14.29	0.005	Slight
Available carbohydrate	23.81	36.73	19.73	0.249	Fair
Sugars	25.17	29.25	18.37	0.117	Slight
Starch	33.33	30.61	12.93	0.000	Slight
Dietary fibre	24.49	35.37	19.05	0.174	Slight
Thiamin	27.21	35.37	13.61	0.029	Slight
Riboflavin	28.57	34.01	14.97	0.009	Slight
Niacin	25.53	43.97	14.89	0.100	Slight
Niacin equivalents	21.09	44.22	14.97	0.743	Substantial
Vitamin C	27.21	34.69	16.33	0.029	Slight
Vitamin E	27.89	35.37	13.61	0.017	Slight
Alpha tocopherol	29.08	32.62	14.89	0.007	Slight
Vitamin B6	24.11	32.62	19.15	0.221	Fair
Vitamin B12	28.37	33.33	16.31	0.013	Slight
Total folate	29.93	34.01	12.93	0.003	Slight
Folic acid	36.05	32.65	8.84	0.000	Slight
Dietary folate equivalents	27.89	37.41	12.24	0.017	Slight
Vitamin A	29.25	33.33	17.69	0.005	Slight
Retinol	31.29	32.65	19.05	0.001	Slight
Beta carotene equivalents	23.13	44.22	15.65	0.344	Fair
Beta carotene	22.70	44.68	14.18	0.422	Moderate
Sodium	25.17	32.65	18.37	0.117	Slight
Potassium	19.05	36.73	17.69	0.772	Substantial
Magnesium	27.21	31.97	12.24	0.029	Slight
Calcium	19.05	40.82	12.24	0.772	Substantial
Phosphorus	23.81	33.33	16.33	0.249	Fair
Iron	26.53	35.37	12.24	0.048	Slight
Zinc	21.09	36.05	19.05	0.743	Substantial
Selenium	34.75	30.50	10.64	0.000	Slight
Iodine	27.89	36.73	14.97	0.017	Slight
VLC N3	30.61	38.10	9.52	0.001	Slight
Linoleic acid	26.53	39.46	15.65	0.048	Slight
Alpha linolenic acid	23.13	44.22	14.97	0.344	Fair
Eicosapentaenoic acid	31.91	36.88	14.18	0.000	Slight
Docosapentaenoic acid	30.50	36.17	13.48	0.002	Slight
Docosahexaenoic acid	29.79	41.13	10.64	0.003	Slight
Caffeine	26.21	36.55	17.24	0.062	Slight
Water	29.25	31.29	19.73	0.005	Slight
Alcohol	44.22	44.22	3.40	0.000	Slight

*n* = 148. ^a^ Gross misclassification = disagreement by three or four quintiles. ^b^ Weighted kappa (κ) measures the degree of disagreement between the nutrient intakes from the two methods. ^c^ Strength of agreement is classified according to weighted κ as follows: 0–0.20 = slight agreement, 0.21–0.40 = fair agreement, 0.41–0.60 = moderate agreement, 0.61–0.80 = substantial agreement [18]. All results reported using log-transformed and energy-adjusted data. Abbreviations: CSIRO, Commonwealth Scientific and Industrial Research Organisation; VLC N3, very long chain n-3 polyunsaturated fatty acids.
